# Emerging Threats in Southern U.S. Pine Plantations: Temporal Dynamics of Fungal Communities and the Impact of *Lecanosticta acicola*

**DOI:** 10.1007/s00248-026-02825-z

**Published:** 2026-07-03

**Authors:** Carolyn R. Cornell, Rabiu O. Olatinwo, Olga Kozhar, Kristi Wharton, Jane E. Stewart

**Affiliations:** 1 Department of Agricultural Biology, Colorado State University, Fort Collins, CO USA; 2 United States Department of Agriculture, Forest Service, Southern Research Station, Pineville, LA USA

**Keywords:** Brown spot needle blight, Fungal pathogens, Loblolly pine, Climate variability, Phyllosphere, Endophytes

## Abstract

Globally, pine forest ecosystems are under increased threat of foliar fungal pathogens. This includes brown spot needle blight (BSNB), caused by *Lecanosticta acicola*. High disease severity of BSNB has been observed in loblolly pine plantations across the Southeastern U.S., causing substantial declines in productivity. Because foliar disease outcomes depend on phyllosphere community interactions, shifts in community composition under climate variation may influence outbreak potential of *L. acicola.* To investigate these interactions, fungal communities in first- and second-year symptomatic and asymptomatic needle tissue were examined over two years across six loblolly pine plantations in central Louisiana. *L. acicola* was consistently enriched in symptomatic needles and emerged as a strong indicator of disease, including increasing crown dieback, particularly in first-year needles. Disease progression was associated with reduced fungal diversity and pronounced shifts in community composition, consistent with microbiome dysbiosis. Additional fungi, including *Lophodermium* and *Soleella*, were enriched in symptomatic needles, likely representing opportunistic associates with a potential role in disease. There were distinct differences in the relationship with climate variables for symptomatic and asymptomatic communities. Symptomatic communities were associated with higher humidity, higher minimum temperatures, and reduced solar radiation, whereas asymptomatic communities were correlated with warmer, drier conditions. Our findings demonstrate that BSNB severity reflects both *L. acicola* infection and broader needle fungal community disruption, with first-year needles being especially vulnerable. These results underscore the need to integrate microbial community dynamics and climate into disease monitoring and management, as increasing humidity, warmer nights, and more variable precipitation likely elevate fungal pathogen risk.

**Supplementary Information:**

The online version contains supplementary material available at https://doi.org/10.1007/s00248-026-02825-z.

## Introduction

Forests, both natural and planted, are complex, diverse ecosystems that provide critical services and valuable renewable resources. In the U.S., plantation forests are continually established and expanded [1] to meet ever-increasing resource demands. Plantation forests in the southern U.S. cover a large portion of the landscape [2], support local economies, and provide the majority of the nation’s timber supply [3]. However, climate variability is expected to significantly alter the structure and function of these forests [4–6], with consequences expected for timber productivity, as well as key ecosystem services such as carbon storage [7] and biodiversity conservation [8]. The reduced tree-species diversity and simplified vertical structure characteristic of plantation forests increase their susceptibility to climate-induced disturbances relative to naturally regenerated forests [9]. Given their ecological, economic, and social importance, forests in the southern U.S. are especially vulnerable, and the impacts of climatic shifts on these systems are of growing concern [10].

Among the many stressors intensified by climate variability, biotic pressures, particularly fungal pathogens, pose an increasing threat to forest productivity [11]. Climate projections of the southern U.S. document that increasing temperatures and variable moisture levels are likely [12], and greater climate variability could create favorable conditions for fungal growth, leading to higher pathogen loads and rising infection risk [13, 14]. This has already been observed for the common forest foliar fungal pathogens *Dothistroma pini* Hulbary and *Dothistroma septosporum* (Doroguine) Morolet [15]. Shifts in climate patterns can alter host–pathogen dynamics by affecting the physiology, development, and interactions of both the host and the pathogen. For instance, pathogens may undergo accelerated evolution, resulting in new, more aggressive strains that are capable of overcoming plant resistance [16, 17]. Climate stressors can also impair normal tree development, from early seedling recruitment to mature growth, reducing host immunity [18] and allowing for less aggressive and endophytic pathogens to successfully cause disease. Additionally, climatic shifts are driving the geographic expansion of both fungal pathogens and their hosts, increasing the likelihood of disease spread into previously unaffected regions [19–21]. As outbreaks intensify, the resulting deterioration in tree health conditions can weaken ecosystem resilience, with far-reaching consequences for affected regions.

Needle blights are fungal diseases contributing to the growing risks to forest ecosystems and have emerged as some of the most serious threats to pine species worldwide. Brown spot needle blight (BSNB), caused by the fungal pathogen *Lecanosticta acicola* (Thümen) Sydow [22], is an important needle disease of *Pinus* species characterized by brown spots within necrotic yellow lesions at infection points and dieback at the distal ends of needles [23]. Severe infection can lead to significant branch loss or even tree death. While *L. acicola* has long been reported to be present in the U.S. [24], it has only recently caused severe dieback in southern U.S. plantations, primarily affecting loblolly pine (*Pinus taeda* L.) [25], the region’s most commercially important forest species [26]. BSNB is also generating global concern with invasive populations being reported in numerous countries [27]. While increased moisture in the form of humidity [28] and/or precipitation [25, 29] is often linked to increased spread of BSNB, information is still limited due to the distinct symptomology on different *Pinus* species [30] and most reported occurrences happening in recent years [31]. Furthermore, climate modeling suggests that *L. acicola* will continue to be a threat in the southern forests over time due to alterations in weather patterns [32]. Significant ecological and economic impacts are now occurring due to BSNB, with spread and severity likely to intensify under current climatic conditions.

For these reasons, we set out to understand the impact of *L. acicola* on loblolly pines grown in monocultures in the southern U.S. by examining the temporal fungal community dynamics in six symptomatic plantations in central Louisiana in October 2023 and 2024. Dynamics focused on the fungal communities inside the needle tissue as *L. acicola* infects *Pinus* species within the needle tissue by entering through the stomata [31]. In this study, we aimed to address: (1) are co-occurring foliar fungi associated with variation in disease severity and progression in the presence of *L. acicola*? (2) how does the fungal community composition vary with the presence of *L. acicola* in first- and second-year needles across collection years?, and (3) is climate variability correlated with variation in *L. acicola* occurrence and tree dieback? The goal was to integrate field-based measurements and molecular approaches to characterize the ecological consequences of recent *L. acicola* outbreaks by examining phyllosphere fungal community responses to infection by assessing how these responses vary with disease severity and climate variability across symptomatic pine plantations.

## Methods

### Sample Collection

Sampling was conducted in Rapides and Vernon Parishes in central Louisiana across six commercial loblolly pine (*Pinus taeda* L.) plantations in collaboration with the U.S. Department of Agriculture, Forest Service, Southern Research Station in Pineville, Louisiana. The six field sites were selected to conduct long-term monitoring to assess loblolly pine crown health and disease severity in plantations with a known presence of brown spot needle blight. Sites were designated as S1, S2, S3, S4, S5, and S6. All sites, except S5, were planted with different CMP families (i.e., full-sib seedlings produced from mass control pollination crosses, also sometimes referred to as CMP). S4 and S5 were planted with half-sib family seedlings produced from uncontrolled crosses, also referred to as open-pollinated. Sites were planted between 2014 and 2018 (Details in Table S1). Central Louisiana has a humid subtropical climate, characterized by generally hot, humid summers and mild winters, with most rainfall occurring in the spring and fall.

In October 2023 and October 2024, ten trees per site were randomly selected, rated for crown dieback, and sampled. All sites exhibited disease symptoms. For each tree, asymptomatic and symptomatic needles were collected from randomly selected branches in the mid-crown. The same trees were resampled across years. Asymptomatic needles were green without observable lesions or necrotic distal portions, while symptomatic needles exhibited visible lesions. First-year (current year) and second-year (previous year) needles were collected from each tree, if present. Needles for DNA extraction were submerged in DNA/RNA Shield (ZYMO Research Corp., USA) in sterile 5 mL tubes immediately after collection. Samples were stored in a cooler and transferred to −80 °C in the laboratory. Needle samples, collected to assess size metrics, disease symptoms, and insect damage, were placed in brown paper bags and stored in a cooler before being transferred to a laboratory and maintained at −20 °C.

### Tree, Needle, and Climate Data Collection

Tree-specific data measured included diameter at breast height (DBH), tree height, and crown dieback level as a measure of disease severity. The crown dieback level was rated on a scale of 1 to 4, with ratings defined as 1 for crowns with < 30% dieback, 2 for 30–60% dieback, 3 for > 60% dieback, and 4 for dead crowns for each tree.

Needle samples from each tree were used to collect size metrics, disease symptoms, and insect damage. For each tree, ten asymptomatic and ten symptomatic needles were randomly selected and examined. This was done for both first- and second-year needles. The lengths of the asymptomatic needles were measured. For symptomatic needles, measurements included length, insect damage, number of lesions, and length of the dead tip. Insect damage was assessed for symptomatic needles as insect wounds may serve as an entry point for the pathogen into the needle tissue.

Weather data were obtained from the PRISM Climate Group (Oregon State University, https://prism.oregonstate.edu) and the Climate Toolbox (https://climatetoolbox.org/) using the geographic coordinates for each site. The variables collected from PRISM included precipitation (precip), minimum temperature (min temp), mean temperature (mean temp), maximum temperature (max temp), minimum vapor pressure deficit (min VPD), maximum vapor pressure deficit (max VPD), and mean dewpoint temperature (dew point temp). Climate Toolbox data included min relative humidity (min RH), max relative humidity (max RH), solar radiation (solar rad), and potential evapotranspiration (evapo). Data consisted of annual averages from 2018 to 2024 (the period between the planting of the last site and sample collection).

## Needle DNA Extraction, Amplicon Sequencing, and Sequence Analysis

DNA from microbes inside the needle tissue was extracted from asymptomatic and symptomatic needles for first-year and second-year needles. DNA was extracted from 385 samples, comprising 164 samples from October 2023 and 221 from October 2024 (Details in Table S2). Samples were generated by pooling needles collected from the same tree to obtain sufficient tissue for extraction. The outside of the needles was sterilized to remove contamination as previously described [33]. In short, needles were washed by vortexing for 10 min in a 0.2% Tween 20 solution, rinsed in 70% ethanol for one min, and dried. Needles were cut into roughly 1 mm long pieces using sterilized tools, and 60 mg of tissue was placed in 2 mL tubes for grinding. For symptomatic needles, lesions and surrounding tissue were excised and used for DNA extraction. Samples were submerged in liquid nitrogen and ground into a powder using the MP Bio FastPrep tissue homogenizer (MP Biomedicals, CA, USA). DNA extraction was performed using the SYNERGY 2.0 DNA Extraction Kit (OPS Diagnostics, NJ, USA) according to the manufacturer’s protocol, with the addition of a chloroform: isoamyl alcohol (24:1) step to remove organic compounds.

The DNA quality and quantity were assessed using a NanoDrop ND-1000 Spectrophotometer (NanoDrop Technologies Inc., DE, USA). For fungal community profiling, the ITS2 region was amplified using the primer set ITS3-2024F (5’-GCATCGATGAAGAACGCAG-3’) and ITS4-2409R (5’-TCCTCCGCTTATTGATATGC-3’). Needle DNA samples were sent to Novogene (Novogene Corporation Inc., CA, USA), where PCR amplification, library preparation, and sequencing were performed. Sequencing was performed on a NovaSeq6000 system (Illumina Inc., CA, USA) using a 2 × 250 paired-end format.

Sequencing reads were returned demultiplexed. Following demultiplexing, Cutadapt [34] was used to remove barcodes and primers from reads, and sequencing data was processed using DADA2 v1.36 [35] in R v4.5.1 [36]. In short, read quality was inspected, and reads were filtered to remove low-quality and short reads using the default expected error settings. Filtered forward and reverse reads were merged using a minimum overlap of 20 nucleotides and zero mismatches. Merged sequences were used to construct an amplicon sequence variant (ASV) table, and chimeric sequences were removed. Taxonomy was assigned using the UNITE database v10 [37]. Non-fungal sequences were removed, and contaminant ASVs were identified and removed using the R package *decontam* [38].

### Data Analysis

Alpha-diversity indices were calculated using mothur [39] by averaging over 1,000 iterations of random subsampling at a depth of 22,360 sequences [40]. Good’s coverage was calculated with mothur, ranging from 99.7% to 99.9% for all samples, to confirm adequate rarefaction depth. Beta-diversity analysis was conducted using R v4.5.1 [36]. The package *metagMisc* [41] was used to randomly subsample and average over 100 iterations at a depth of 22,360 sequences to create an ASV table for beta-diversity calculations. Bray-Curtis taxonomic beta-diversity was calculated using *vegdist* in *vegan* [42]. Weighted UniFrac phylogenetic beta-diversity was calculated using *GUniFrac* [43] using a phylogenetic tree constructed in QIIME2 v2025.4 [44]. Relative abundance analysis of microbial taxa was performed using the R package *phyloseq* [45].

Statistical analyses were performed in R v4.5.1 [36]. Principal component analysis (PCA) was used to visualize climatic gradients across study sites and examine the relationship between tree crown dieback and needle phenotypes. Weather differences across sampling years were assessed using fold change (log_2_), and significant differences were determined via ANOVA. Linear mixed-effects models (LMMs) were used to assess relationships among tree phenotypes, crown dieback, sampling year, and planting year, with site and tree included as nested random effects. Estimated marginal means and trends were calculated using the *emmeans* package [46], with Tukey-adjusted p-values used for pairwise comparisons. Using the same random-effects structure, LMMs were also used to determine the impacts of symptom status, sampling date, and needle year on alpha-diversity indices across the full dataset, with additional models conducted separately within each needle year. Models were also fitted with crown dieback substituted for symptom status. Beta-diversity patterns, to determine differences in community structure by symptom status and dieback level, were visualized with 3D principal coordinate analysis (PCoA) using *vegan3d* [47]. Dispersion within groups was assessed, and the statistical significance of main factors on beta-diversity was evaluated using PERMANOVA via *adonis2* in *vegan* [42]. Pairwise multilevel comparisons using adonis were conducted to compare dieback levels (*P* < 0.05) using the *pairwiseAdonis* package [48], accounting for repeated measures on trees. P-values were adjusted using the Benjamini–Hochberg (BH) method. For differential abundance analysis between symptom status, the linear discriminate analysis of effect size (LEfSe) method was employed using the package *microbiomeMarker* [49], with p-values adjusted using the BH method. The significance level was set to 0.05, and the threshold was set to 3.5. Fungi associated with symptom status and dieback level were identified using the *indicspecies* package in R [50]. Network analysis, based on Pearson’s correlations, was used to assess the strength and direction of the relationship between weather factors and the Shannon diversity of first-year needles, using *igraph* [51]. Only significant correlations (*P* < 0.05) were included in the network. Significant correlations were then plotted and fitted with a linear model to determine whether symptom status or sampling data explained the relationships in alpha-diversity. Climatic factors were fitted to the beta-diversity PCoA to examine the direction of the relationships with symptom status using *envfit* in *vegan* [42]. The first PCoA axis was extracted and compared to the climatic factors using linear regression.

## Results

### Climate, Tree, and Needle Trait Variation Across Diseased Loblolly Plantation Sites

**Fig. 1:**
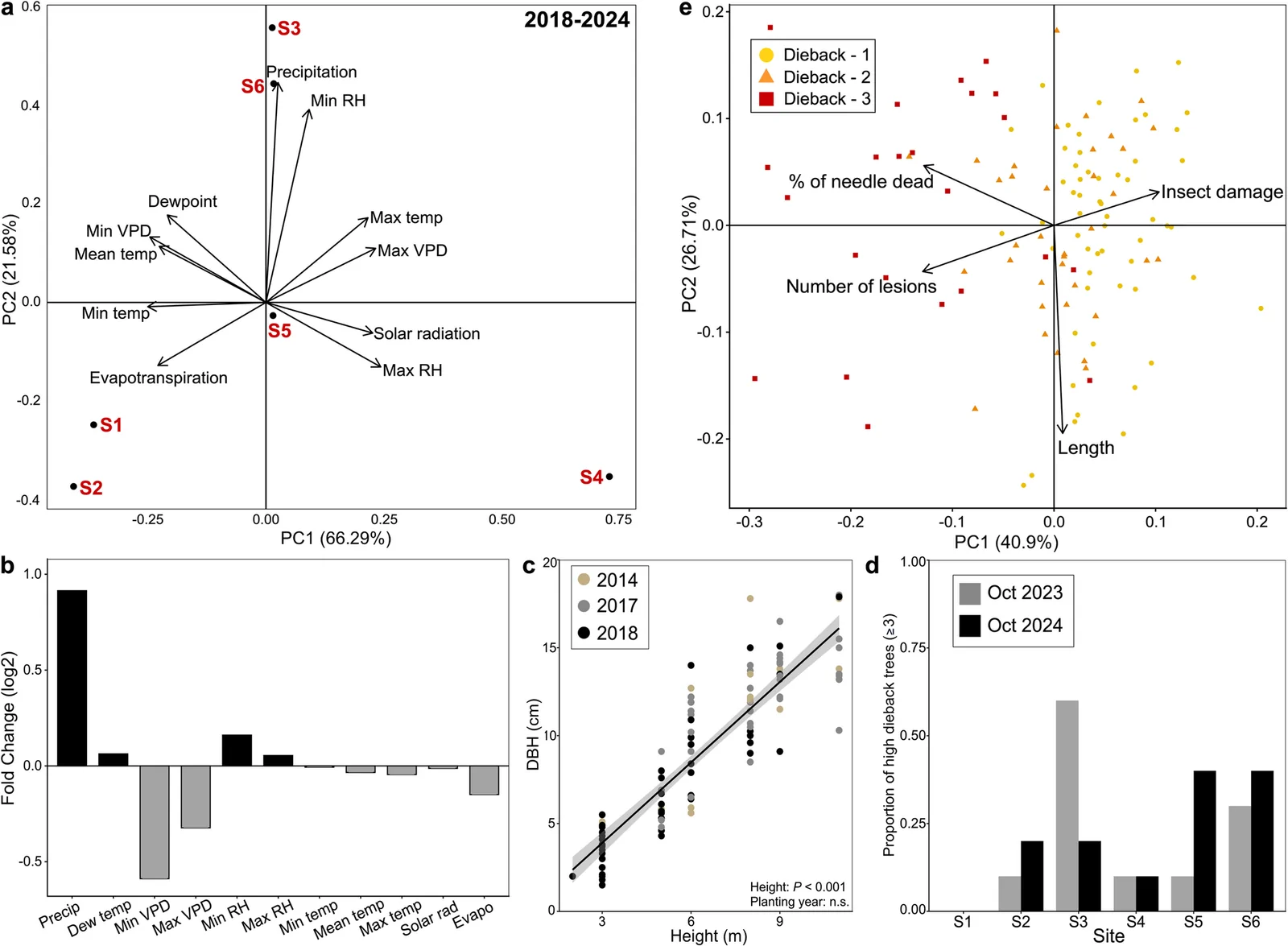
Climate, tree, and needle trait differences across diseased sites. (**a**) Comparisons of climate variables across six study sites based on principal component analysis (PCA). Climate data based on yearly averages from the planting of the last site (2018) until final sample collection (2024). (**b**) Fold change of weather variables between October 2023 and October 2024 sample collection. October 2023 was used as the baseline. (**c**) The linear relationship between tree height and diameter at breast height (DBH). Points represent individual trees, with color by planting year: tan for 2014 (S1), gray for 2017 (S3, S6), and black for 2018 (S2, S3, S4). Significant relationships based on linear mixed-effects model, with site and tree included as nested random effects. Non-significant relationship are abbreviated as n.s. (**d**) Proportion of sampled trees at each site exhibiting high crown dieback (≥ 3) in October 2023 (gray) and October 2024 (black). (**e**) PCA of needle property data and tree crown dieback level for first-year needles, with crown dieback 1 being low defoliation (< 30%) and crown dieback 3 being high defoliation (>60 %). High dieback corresponded to disease symptoms in needles (i.e., number of lesions and needle death)

Notable climatic differences were observed across the six loblolly field sites from planting to needle sampling (Fig. 1a). Long-term averages of the standardized precipitation index (SPI) at 1-year timescales indicated sites were generally wetter than normal since planting, with study sites experiencing extremely wet (> 2.00) and very wet (1.50 to 1.99) conditions between 2020 and 2023 with noticeable intermittent drought, followed by a pronounced severe drought (−1.99 to −1.50), and extreme drought (< −2.00) in 2023 (Fig. S1). Sites S3 and S6 (Rapides Parish) experienced the greatest cumulative precipitation (~ 12,408 mm from 2018 to 2024) and higher min RH. In comparison, sites S1 and S2 (Vernon Parish) had the least precipitation (11,087 mm from 2018 to 2024) and the highest min temp, mean temp, and evapotranspiration, on average. Weather conditions also differed between the 2023 and 2024 sampling years (Fig. 1b). All variables, except min temp, significantly (*P* < 0.05) differed between years, with the largest contrast occurring in moisture-related variables. Overall, 2024 was characterized by wetter, cooler conditions than 2023, underscoring interannual variability and precipitation gradients across sites.

Trees displayed variations in properties, including crown dieback (a measure of disease severity), height, and DBH. Taller trees exhibited significantly greater DBH (*P* < 0.001; Fig. 1c), and both increased from 2023 to 2024 (*P* < 0.05). Despite planting dates occurring from 2014 to 2018, the tree size of S1, planted in 2014, did not differ significantly from all other sites planted in 2017 and 2018. Tree height and DBH also decreased with increasing levels of crown dieback (Fig. S2a). Height exhibited a consistent negative relationship with dieback (*P* < 0.05), whereas DBH showed a stronger negative relationship with dieback in 2024 (*P* < 0.001). Average crown dieback varied among sites and across sampling years (Fig. S2b). In 2023, sites S3 and S6 had the greatest crown dieback, reflected in a high proportion of trees with substantial crown loss (≥ dieback 3; Fig. 1d). In 2024, the proportion of trees with high crown dieback rose in sites S2, S5, and S6 (Fig. 1d).

Needle disease symptoms aligned with crown dieback patterns observed in the trees across sites (Fig. 1e). Trees exhibiting greater crown dieback levels showed greater disease severity in needles, with level 3 dieback being associated with more BSNB lesions and a greater portion of each needle exhibiting distal necrosis. Insect wounds were not associated with increased disease severity, suggesting they were likely not a primary entry point of the pathogen into the needle tissue in these sites.

### Needle Fungal Community Diversity Variation by Symptom Status and Crown Dieback Level

**Fig. 2:**
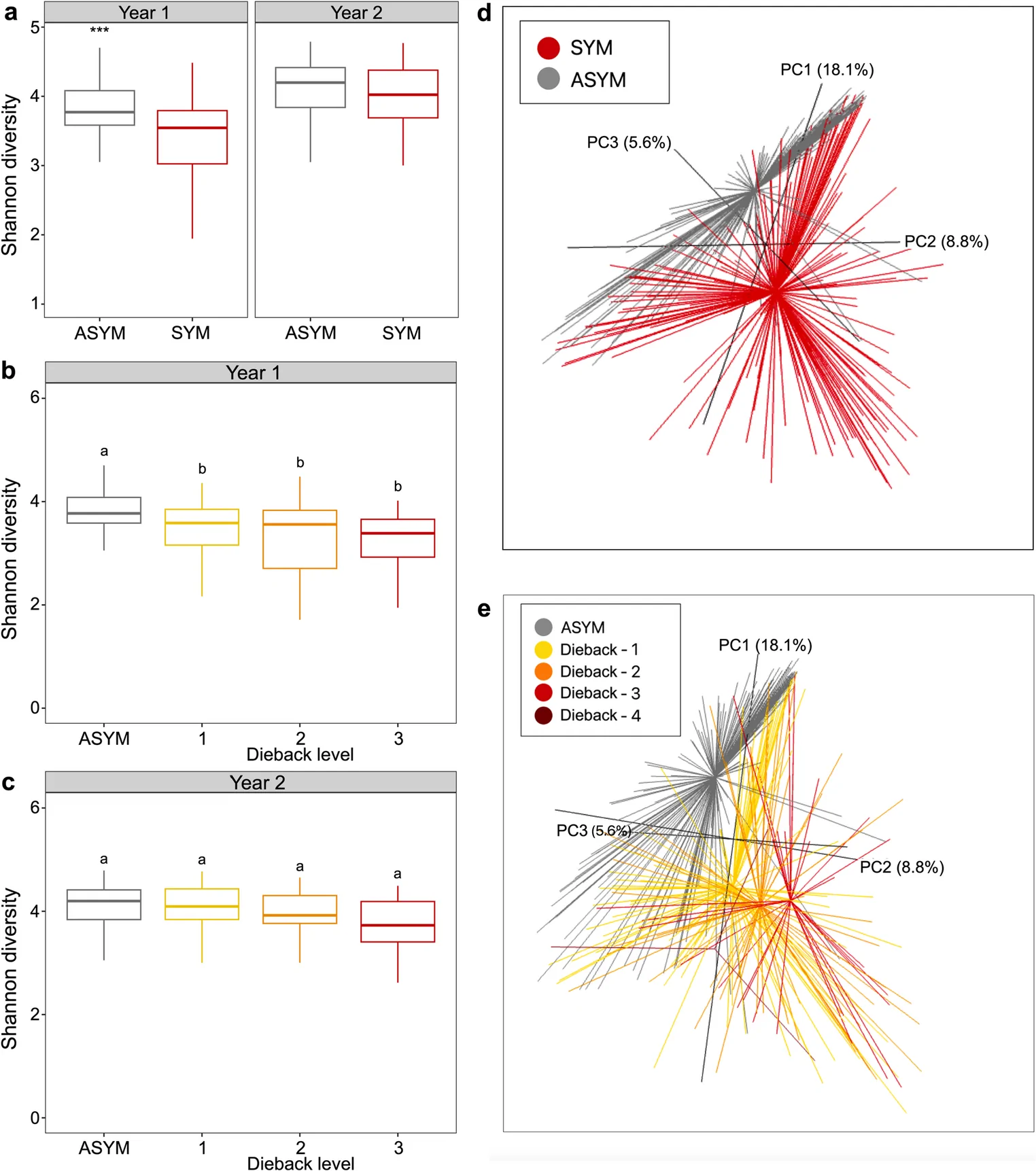
Fungal needle diversity by symptom status and dieback level. Alpha diversity, based on Shannon diversity index, by (**a**) symptom status for first- (Year 1) and second-year (Year 2) needles. Shannon diversity of (**b**) first- and (**c**) second-year needles by dieback level. Crown dieback 4 (dead) samples were excluded from analysis due to the small sample size. Groups sharing the same letter were not significantly different. Significance is expressed as ***P < 0.001. 3D principal coordinate analysis (PCoA) of Bray-Curtis dissimilarity by (**d**) symptom status and (**e**) crown dieback levels. Asymptomatic abbreviated as ASYM, and symptomatic abbreviated as SYM

Alpha-diversity indices, including Shannon diversity and richness, were influenced by needle year (first-year vs. second-year), symptom status (asymptomatic vs. symptomatic), and crown dieback level. Diversity (Fig. 2a) and richness (Fig. S3a) were significantly lower (*P* < 0.01) in symptomatic needle communities compared to asymptomatic communities for first-year needles, whereas there was no significant difference by symptom status for second-year needles. This pattern was consistent across sampling dates, but the magnitude was greater in Oct 2024, particularly for richness. Similarly, diversity (Fig. 2b) and richness (Fig. S3b) in first-year needles were significantly lower across all dieback levels compared to asymptomatic communities (*P* < 0.001 and *P* < 0.05, respectively), with richness declines driven primarily by differences in Oct 2024. Mean diversity and richness continued to decline with increasing crown dieback, although differences among dieback levels were not significant. Again, no significant effect of crown dieback levels was detected in second-year needles (Fig. 2c; Fig. S3c), despite similar downward trends in mean diversity and richness. This indicates that symptomatic declines in alpha-diversity were largely a signature of first-year needles.

Building on these patterns, beta-diversity also exhibited clear differences by symptom status and crown dieback level. When examining the needle community structure as a whole, asymptomatic and symptomatic needle communities formed distinctly clustered communities (Fig. 2d). This pattern was evident in first- and second-year needles (Fig. S3d). Symptom status was consistently the strongest predictor of shifts in community structure (Table 1), followed by needle year and sampling date (Fig. S3e) for taxonomic and phylogenetic beta-diversity. Site explained a large portion of the variation, but showed a comparatively weaker effect.

**Table 1:** Significance test of needle communities based on PERMANOVA

	Main factors	df	R^2^	F	*P*
Taxonomic	Symptom	1	0.052	23.98	0.001
	Needle year	1	0.042	19.44	0.001
	Sampling date	1	0.015	6.86	0.001
	Site	5	0.069	6.50	0.001
Phylogenetic	Symptom	1	0.110	58.06	0.001
	Needle year	1	0.060	31.45	0.001
	Sampling date	1	0.018	10.46	0.001
	Site	5	0.077	8.24	0.001

Model structure: dissimilarity matrix ~ Symptom * Needle year * Sampling date + Site

Permutations constrained within repeated measures (strata = Tree)

Interaction terms were tested, but only main effects were included in the table

The needle fungal community structure also progressively shifted as crown dieback level increased (Fig. 2e), with all dieback levels significantly differing from asymptomatic communities (*P* < 0.01). Additionally, lower crown dieback (levels 1 and 2) communities differed from high dieback communities (level 3; *P* < 0.01) taxonomically, but only moderately phylogenetically (*P* < 0.1). The overall changes in community structure were consistent across sampling years and were strongly associated with symptom development and the extent of crown dieback.

### Fungal Community Dynamics and the Roles of *Lecanosticta* in Tree Crown Dieback

**Fig. 3:**
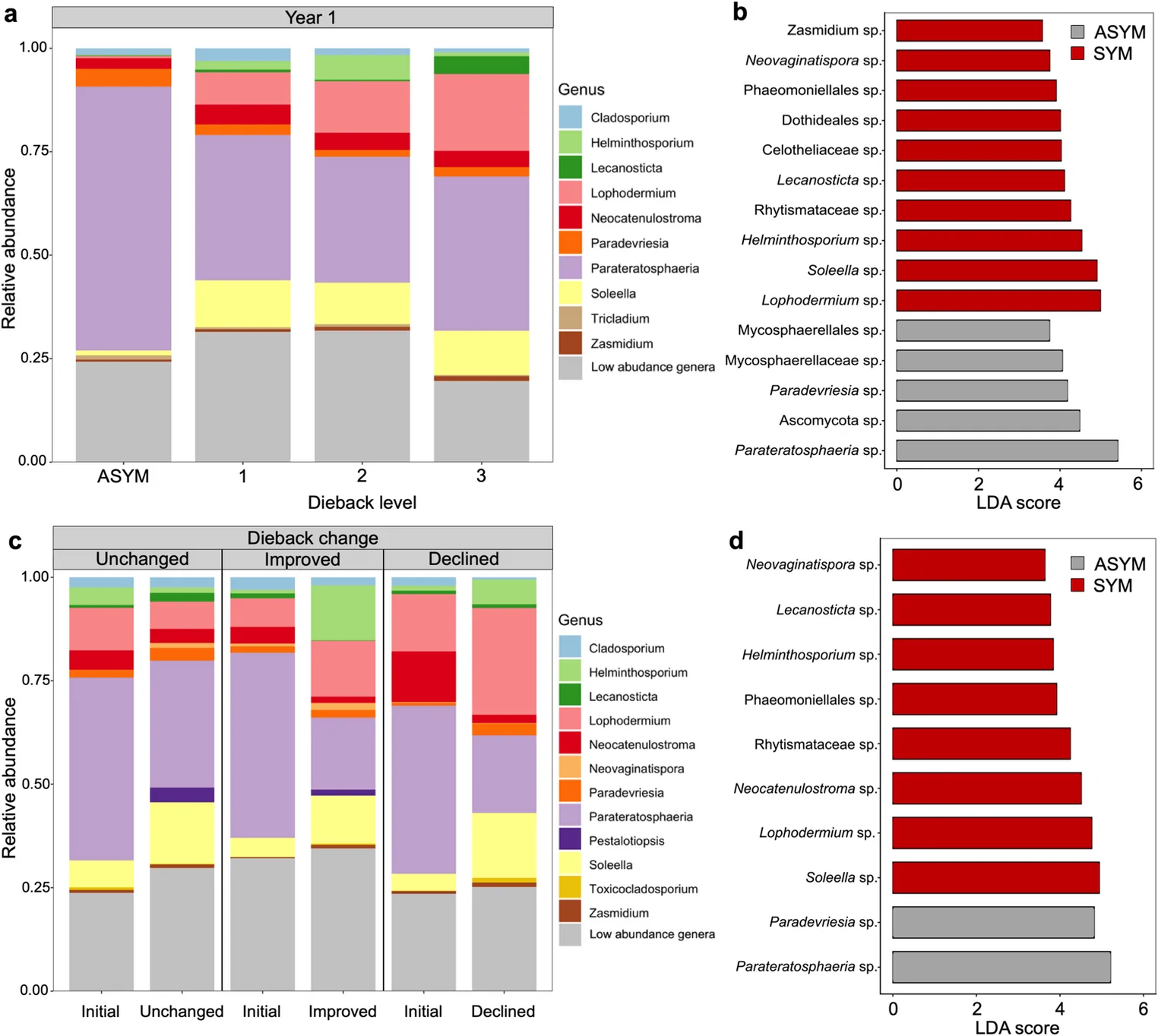
Differences in the relative abundance of abundant fungal genera. (**a**) The relative abundance of dominant fungal genera by crown dieback level in first-year needles. (**b**) Differential abundance analysis between symptom status (ASYM vs. SYM) of first-year (Year 1) needles using LEfSe (Kruskal–Wallis, BH adjusted *P* < 0.05). Linear discriminant analysis (LDA) threshold was set at 3.5 or greater. (**c**) The change in relative abundance of dominant fungal genera in symptomatic first-year needles. Samples are grouped by whether dieback levels remained unchanged (*n* = 39), improved (*n* = 8), or declined (*n* = 13) between initial sampling in October 2023 (initial) and October 2024. (**d**) Differential abundance analysis between symptom status of second-year needles using LEfSe (Kruskal–Wallis, BH adjusted *P* < 0.05). Linear discriminant analysis (LDA) threshold was set at 3.5 or greater. Asymptomatic abbreviated as ASYM, and symptomatic abbreviated as SYM

Fungal communities in needle tissues differed between asymptomatic and symptomatic needles, with the relative abundance of *Lecanosticta* increasing as crown dieback progressed in trees. This was observed in first- (Fig. 3a) and second-year needles (Fig. S4). Although *Lecanosticta* occurred at all sites, it was rare in S1 communities (< 0.1%). In addition to *Lecanosticta*, several other fungal genera were also significantly more abundant (*P* < 0.05) in symptomatic communities (Fig. 3b and d), particularly *Lophodermium* and *Soleella*. The relative abundance of *Soleella* remained constant across crown dieback levels, yet *Lophodermium* increased with increasing crown dieback. In contrast, asymptomatic needle communities were dominated by the genera *Parateratosphaeria* and *Paradevriesia*. Differential abundance analysis confirmed that these genera were significantly enriched (*P* < 0.05) in asymptomatic first- (Fig. 3b) and second-year (Fig. 3d) needles. These community-level differences in relative abundance were consistent across sampling dates (Oct 23 and Oct 24). While the dieback level remained unchanged for most trees, a subset exhibited improvement or decline between sampling years (Fig. 3c, Table S3). Specifically, for first-year needle communities, *Lecanosticta* increased in relative abundance from October 2023 to October 2024 for trees that remained unchanged or declined, whereas *Lecanosticta* decreased in trees that showed improvement.

**Fig. 4:**
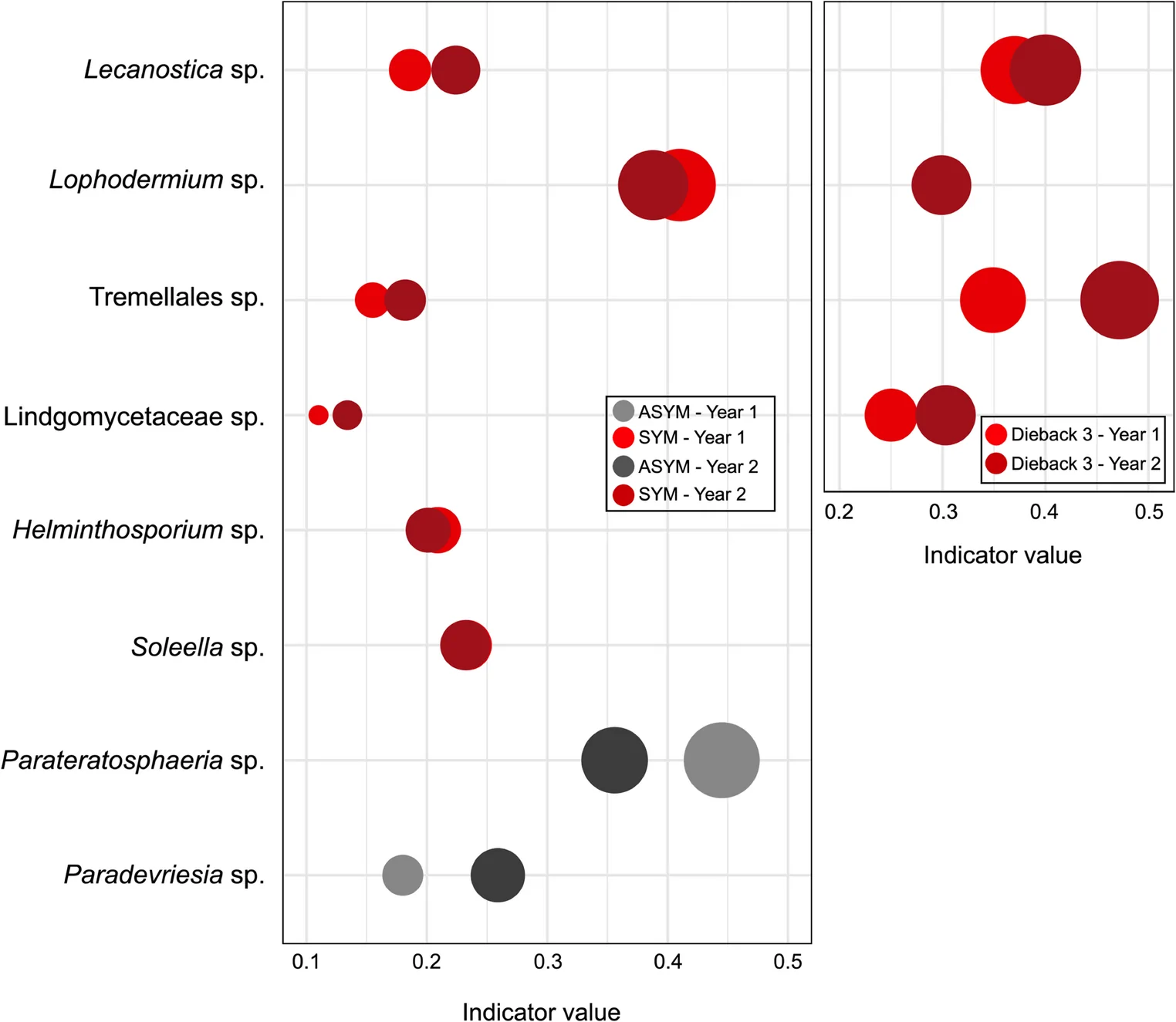
Indicator species of symptomatic and high dieback fungal communities. Fungal indicator species (ASVs) of asymptomatic (ASYM) and symptomatic (SYM) needle communities of first- (Year 1) and second-year (Year 2) needles. Indicator species of needle communities of trees with high crown dieback (dieback 3) also shown. All indicators were significantly associated with their respective groups (*P* < 0.05). The size of the points also represents indicator values on the x-axis. The lowest level of taxonomic classifications is shown for each indicator

Since relative abundance patterns revealed distinct shifts in community structures across symptom status and crown dieback levels, indicator species analyses were conducted to determine which taxa served as the strongest markers of these conditions. ASVs belonging to *Lecanosticta* and abundant genera were identified as indicators of symptomatic needle communities (Fig. 4). *Lecanosticta*, *Lophodermium*, and *Soleella* species were indicators of first- and second-year symptomatic needle communities. The *Lecanosticta* ASV was a 100% match (in terms of query coverage and percent identity) to the expected pathogen, *Lecanosticta acicola*. Additionally, the *Lophodermium* indicator matched *Lophodermium australe* (100% query coverage and percent identity), and the *Soleella* indicator could not be assigned to a species based on BLAST results. In comparison, when examining indicators of high crown dieback communities, *Lecanosticta* was the strongest indicator of first-year needles and a significant indicator of second-year needles. *Lophodermium* was not an indicator of high crown dieback in first-year needles but became significant in second-year needles. These findings support that *Lecanosticta* was the primary driver of disease in first-year needles; however, a complex interplay between the fungal pathogen and other fungi, such as *Lophodermium*, may contribute to progression in crown dieback, especially in second-year needles.

### Differential Weather Responses of Symptomatic and Asymptomatic Fungal Communities

**Fig. 5:**
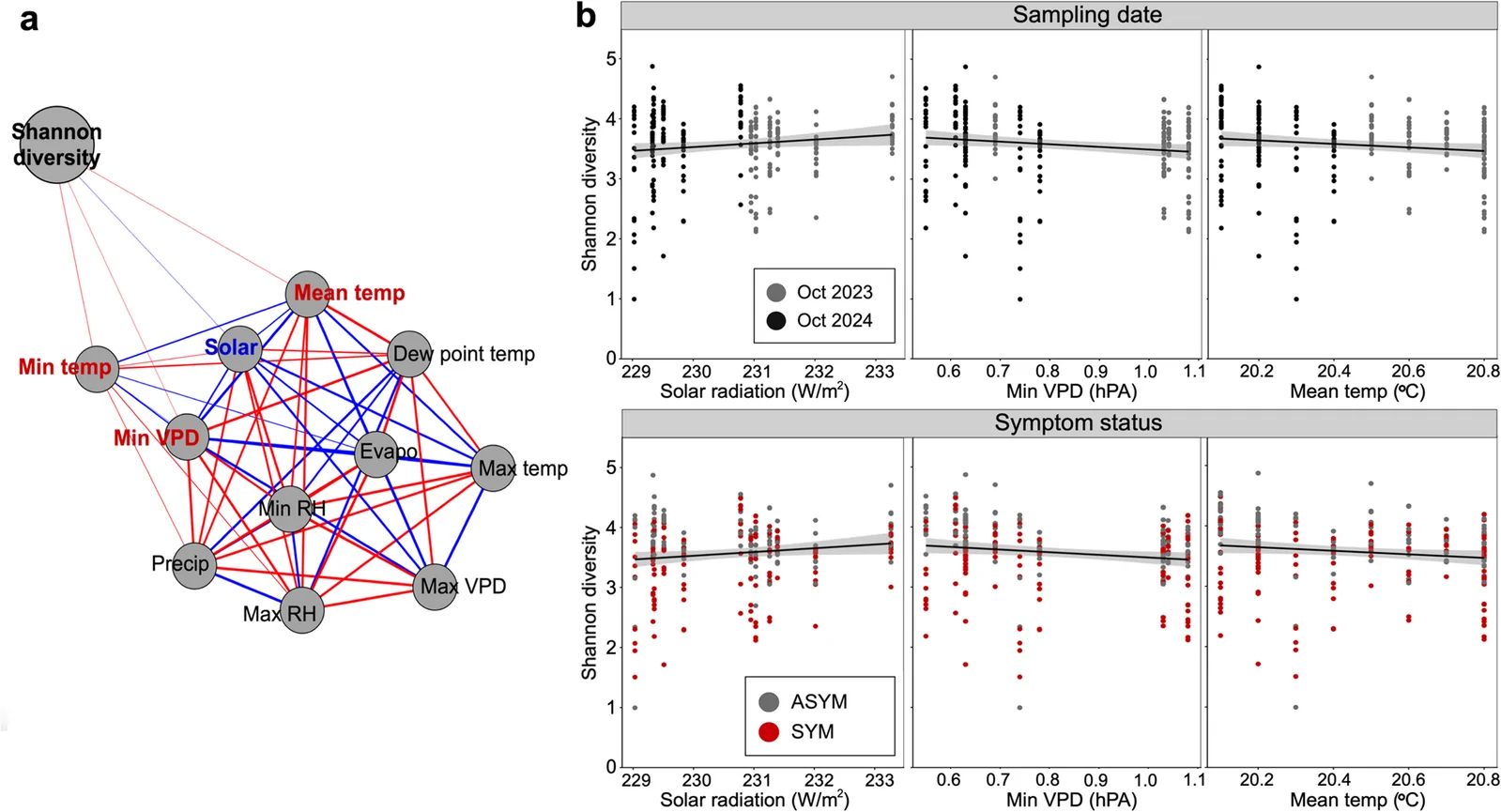
Impact of climatic factors on Shannon diversity of first-year needle communities. (**a**) Network analysis of Shannon diversity and climatic factors based on Pearson’s correlations (*P* < 0.05). Factors directly correlated with Shannon diversity are shown in blue and red, with blue representing positive correlations (*r* > 0) and red representing negative correlations (*r* < 0). Line thickness depicts the strength of the correlations. (**b**) Drivers of the relationship between Shannon diversity and directly correlated climatic factors. Sampling date (October 2023 and October 2024) and symptom status (ASYM and SYM) appeared to be the main drivers, depending on the factor. Asymptomatic abbreviated as ASYM, and symptomatic abbreviated as SYM

Fungal Shannon diversity varied with weather conditions, largely driven by differences in responses related to symptom status and variations between sampling dates (Fig. 5). Shannon diversity was significantly (*P* < 0.05) negatively correlated to min VPD, min temp, and mean temp, while solar radiation showed a significant positive correlation (Fig. 5a). For symptom status, the pattern was similar across all variables. The diversity of asymptomatic communities generally fell above the regression trend lines, indicating a higher-than-expected diversity and potentially a more resilient response to weather variation relative to symptomatic communities. Sampling year also had a strong influence on diversity in relation to mean temp, minimum vapor pressure deficit (minVPD), and solar radiation (Fig. 5b). In general, higher temperatures and drier conditions (higher min VPD) were associated with reduced fungal diversity, reflecting weather differences between October 2023 and October 2024.

**Fig. 6:**
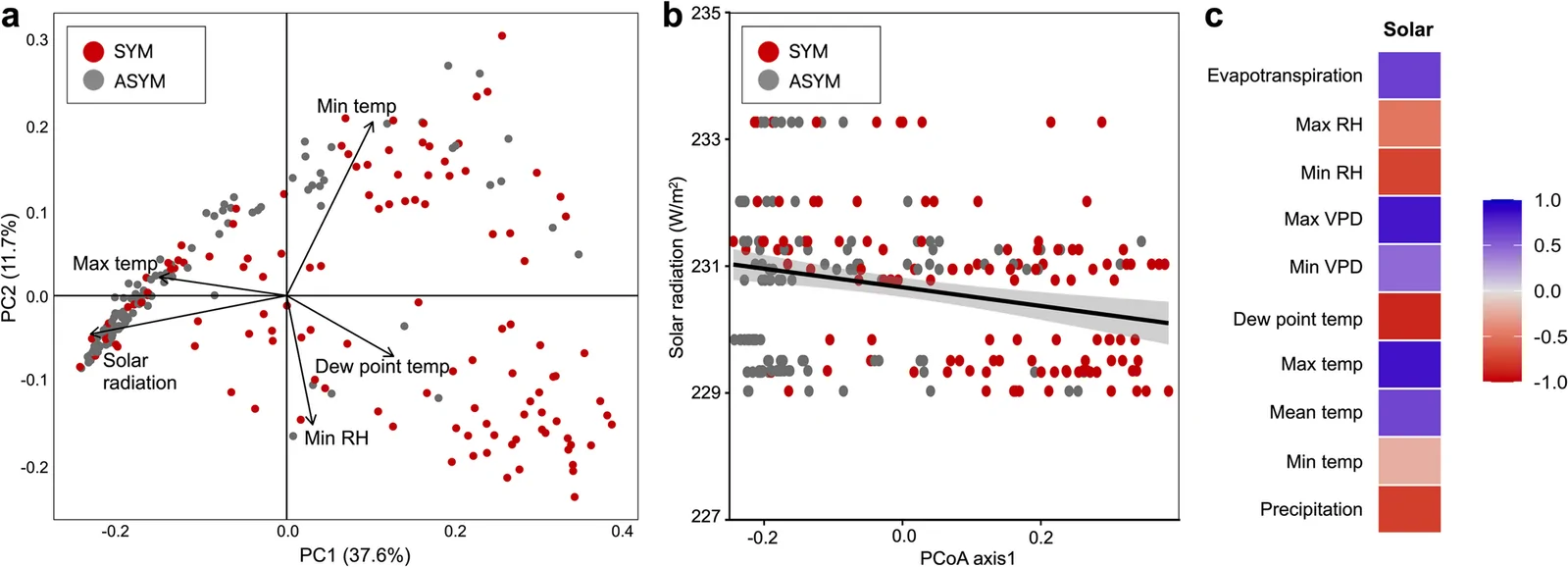
Influence of climatic factors on first-year needle community beta-diversity. (**a**) Principal Coordinates Analysis (PCoA) of needle community composition based on weighting UniFrac dissimilarity. Climatic factors correlated with the ordination (*P* < 0.1) are shown as vectors with highly correlated variables removed. (**b**) Correlation between the first PCoA axis (weighting UniFrac) and climatic factors using linear regression. The strongest correlation to the first axis, based on the R^2^ value, is shown. (**c**) Relationship between other climatic factors and solar radiation based on Pearson’s correlations. Asymptomatic abbreviated as ASYM, and symptomatic abbreviated as SYM

Moisture and temperature variables also explained patterns in beta-diversity across symptom status (Fig. 6a). Asymptomatic communities were associated with higher solar radiation and max temp, whereas higher min RH, dew point temp, and min temp were linked with symptomatic communities. Solar radiation had the strongest correlation with the first PCoA axis (Fig. 6b), with higher solar radiation corresponding with asymptomatic communities and lower solar radiation associated with symptomatic communities. As expected, solar radiation was positively correlated with max temp and negatively correlated with min temp, dew point temp, min RH, and max RH (Fig. 6c). These relationships indicate that warmer, drier conditions favor asymptomatic communities, whereas more humid conditions and likely elevated minimum temperatures create favorable conditions for the BSNB pathogen and symptomatic communities.

## Discussion

Over the last decade, the presence of *Lecanosticta acicola* and the associated disease, brown spot needle blight, has expanded across the Northern Hemisphere, becoming one of the most serious needle diseases of *Pinus* species [52]. Yet, little is still known about how needle fungal communities in the presence of *L. acicola* change over time and with needle age. Therefore, we examined infected trees across six loblolly pine plantations to better understand how *L. acicola* alters needle-associated fungal community dynamics at varying levels of crown dieback. Our results suggest that needle disease severity is associated with the BSNB primary pathogen, *Lecanosticta acicola*, and additional fungal associates whose contributions vary with disease progression and needle age. *Lecanosticta* was consistently enriched in symptomatic needles and was a strong indicator species of both symptom status and higher levels of crown dieback. Initial findings also indicated that varying dieback conditions across years corresponded with changes in *Lecanosticta* relative abundance. However, disease progression may not be monocausal, as other fungi, including *Lophodermium* and *Soleella* species, were also enriched in symptomatic needles, with *Lophodermium* increasing with crown dieback. Similar studies also found other taxa associated with BSNB, including *Lophodermium*, implicating possible interactions [53, 54]. While some *Lophodermium* species are causal pathogens of needle cast diseases [55], our sequences were most similar to saprotrophic species based on BLAST identification and these taxa were also detected in asymptomatic needle communities, suggesting potentially a saprophytic role. These associated saprophytic taxa are likely to benefit from tissue compromised by *L. acicola* than to function as secondary pathogens. However, it cannot be ruled out that this or other taxa may be pathogenic, as ITS data alone does not provide sufficient resolution to distinguish among closely related species. Considering that plant pathogens often work in complexes to cause disease [56], it is possible that *L. acicola* interacts or coexists with other species that play a role in disease progression.

The influence of *Lecanosticta* and fungal associates is not uniform across the host. In particular, the needle age of loblolly pines was a critical determinant in shaping the interactions among disease, the host, and the fungal community. As BSNB has continued to spread across continents and *Pinus* species [57], *L. acicola* has often been observed on first-year needles [58, 59]. Consistent with this trend, first-year loblolly needles in our study exhibited a decline in alpha-diversity with disease onset, whereas second-year needle diversity remained relatively stable regardless of symptom status. Younger needles tend to have lower concentrations of defensive secondary plant compounds [60–62], making them more susceptible to pathogen invasion and disease-driven community disruption. Disease symptoms on the second-year needles likely represent carryover infection acquired the previous year. The dynamics in second-year needles may signify that the fungal community has already been filtered due to changes in characteristics with needle aging [63], priority effects [64], and opportunistic taxa colonizing damaged tissue [65]. Together, these results highlight the heightened vulnerability of first-year loblolly needles to pathogen invasion, with likely cascading effects on fungal community structure.

Declining fungal diversity of needle tissue communities emerged as a consistent feature associated with disease symptoms, resulting in microbiome dysbiosis, in which the community becomes unbalanced in structure or function due to pathogen presence [66, 67]. BSNB symptomatic needle communities experienced reduced alpha-diversity, as well as prominent shifts in structure with increased tree crown dieback. Similar dysbiosis patterns have been observed in other plant leaf microbiomes, with alterations in microbiome composition, richness, and abundance associated with pathogen presence [68–70]. Conversely, higher microbial diversity in leaf communities is frequently coupled with greater host health [71] by maintaining host homeostasis and enhancing defense against microbial intruders [72, 73]. The asymptomatic needle communities in this study followed this pattern, harboring more diverse and structurally distinct fungal communities with little to no presence of *Lecanosticta*. Since *Lecanosticta* was generally absent from asymptomatic fungal communities and resides on the needle surface before entering the tissue [74], it is likely that *L. acicola* invades and disrupts the endophytic community, contributing to the observed structural shifts. *L. acicola* did not competitively exclude community members based on abundance, but possible functional dominance remains a plausible mechanism [75]. Additionally, tissue necrosis caused by BSNB likely altered niche availability [76], changing the fungal diversity and structure of the needle tissue community. This is supported by the shift in fungal diversity and greater prevalence of saprophytic fungi with increasing crown dieback, consistent with a move towards communities adapted to decomposing necrotic tissue. These patterns suggest that BSNB-associated disease progression is closely linked to the disruption of needle fungal communities, potentially allowing external environmental factors, including climate, to further influence host–pathogen interactions. Additionally, climate-related stressors may impact host physiology and alter microbiome stability, thereby facilitating *L. acicola* establishment, although this needs further investigation.

Climate is a critical factor affecting plant disease development, including the severity of brown spot needle blight [57]. Disentangling the impact of climatic factors on plant disease remains challenging, as they can have both direct and indirect effects on the pathogen, plant, and plant-pathogen interactions [17]. For BSNB, there were distinct differences in the correlations with climate variables for symptomatic and asymptomatic communities. Symptomatic needle tissue communities were associated with higher moisture levels, higher minimum temperatures, and reduced solar radiation. Moisture variables, including precipitation [77], humidity [29, 30], and dew point temperature [78], have all been previously shown to influence *L. acicola* disease occurrence. This is anticipated as moisture is required for spore germination, mycelial development, host stomatal penetration, fructification body formation, and spore production and release [52] in the lifecycle of *L. acicola* and other fungal pathogens [79, 80]. Conidia germination and release are also temperature-dependent [31, 81, 82]. Higher minimum temperatures promote these processes and could favor *L. acicola* infection by extending periods conducive to conidial viability and dispersal. Similar trends have been observed with warmer or milder winters increasing *L. acicola* spread [83, 84]. In comparison, asymptomatic communities were linked to drier conditions with higher air temperatures and increased solar radiation, which may limit disease spread. This differs from other studies, which found higher spore counts associated with greater daily maximum temperatures [29, 52]. However, elevated temperatures combined with dry conditions have been suggested to constrain the spread of *L. acicola* [32]. Increased solar radiation is likely to have multiple effects, as it is closely linked to several variables (e.g., light, temperature, humidity). For example, increased light (higher solar radiation) stimulates stomata opening [82], whereas drier conditions generally induce stomatal closure, having opposite effects on the entry of *L. acicola* into needle tissue via the stomata. Elevated UV exposure has also been observed to inactivate several fungal pathogens on leaf surfaces [85, 86]. Therefore, while increased solar radiation was associated with asymptomatic communities, reduced disease presence is likely due to a complex interplay between the responses of the pathogen and the host to climate factors. Additionally, observed interannual variability across sites likely further altered host-pathogen interactions by altering the climatic conditions that influence disease dynamics. Our findings are consistent with current climate trend predictions, showing an increasing risk of fungal pathogen infection under higher humidity, warmer nights, and more variable precipitation patterns. Importantly, these results extend current understanding of *L. acicola*, which has primarily been derived from spore-based studies, by demonstrating how climate variability is associated with shifts in naturally occurring needle fungal community composition during disease expression.

## Conclusion

Given the importance of southeastern U.S. loblolly plantations to national forestry [87] and the continued global spread of brown spot needle blight, it is critical to understand disease dynamics beyond spore release. In this study, fungal needle communities in loblolly pine plantations were strongly structured by *Lecanosticta acicola*, especially in first-year foliage; however, our results indicate that disease severity and increasing crown dieback reflect broader disruptions to needle fungal communities, possibly through interactions with other opportunistic non-pathogenic fungi. In loblolly pines, *L. acicola* infection likely reflects the invasion of young, vulnerable needles before stable fungal community formation, with cascading consequences for community composition and host health. The loss of fungal diversity and transition toward saprotroph-dominated communities suggest a shift from symbiotic or neutral interactions toward communities structured by tissue necrosis and resource availability. Climate further influences these dynamics by shaping both pathogen activity (e.g., stomatal entry, spore release) and host susceptibility, reinforcing the idea that disease expression arises from complex interactions among microbes, hosts, and the environment. While our study provides strong evidence linking *L. acicola*, fungal community structure, and climate to BSNB severity, further research is needed. In the laboratory and greenhouse, experimental inoculations, environmental manipulation, and functional studies, combined with long-term field monitoring, could help resolve causal mechanisms, determine the importance of species interactions, and assess the stand-level impacts of repeated infection cycles.

Early detection of BSNB is likely critical for maintaining stand health. Our results indicate that monitoring efforts should prioritize first-year loblolly needles, symptom expression and disease progression were more apparent in first-year needles. *L. acicola* spreads easily within a stand via rain splash [57], and reducing stand density may help limit transmission by reducing needle proximity. Thinning practices that increase light penetration may provide further benefits, as higher solar radiation was consistently associated with asymptomatic needle communities. Increased light penetration and a greater distance between trees could reduce moisture retention and relative humidity, thereby reducing favorable conditions for *L. acicola*, while also alleviating inter-tree competition. Beyond stand structure, our findings suggest that maintaining diverse needle tissue-associated fungal communities may help maintain needle health. The direct management of fungal diversity is challenging; therefore, practices that minimize disturbance to microbial communities during periods of high vulnerability (e.g., stress) could help preserve diversity. Mixing genetics within a single species plantation may also represent another method to enhance host resistance, microbial diversity, and disease resilience. Genotypes were not replicated across sites in this study, therefore, potential genetic effects could not be disentangled from site-specific environmental conditions or stand age. Notably, the healthiest plantation was also the oldest stand, introducing additional confounding among genotype, site conditions, and plantation age. The effective management of BSNB will likely depend on strategies that address host dynamics, pathogen dispersal, and the maintenance of resilient needle microbial communities.

## Supplementary Information

Below is the link to the electronic supplementary material.

Supplementary Material 1 (DOCX 9.78 MB)

## Data Availability

ITS gene sequences were deposited in the Sequence Read Archive (SRA) under the BioProject number PRJNA1431429.
