# Supplementary material for: Emerging Threats in Southern U.S. Pine Plantations: Temporal Dynamics of Fungal Communities and the Impact of Lecanosticta acicola
Source: Microb Ecol. 2026 Jul 3;89:177. doi: 10.1007/s00248-026-02825-z (PMC13612803; doi:10.1007/s00248-026-02825-z)
Supplement: Supplementary file 1 [file 248_2026_2825_MOESM1_ESM.docx]

**Emerging Threats in Southern U.S. Pine Plantations: Temporal Dynamics of Fungal Communities and the Impact of *Lecanosticta acicola***

Carolyn R. Cornell^a,1^, Rabiu O. Olatinwo^b^, Olga Kozhar^a*^, Kristi Wharton^b^, Jane E. Stewart ^a,1^

^a^Department of Agricultural Biology, Colorado State University, Fort Collins, Colorado, USA

^b^United States Department of Agriculture, Forest Service, Southern Research Station, Pineville, Louisiana, USA

Carolyn R. Cornell: https://orcid.org/0000-0001-5355-3248

Rabiu O. Olatinwo: https://orcid.org/0000-0002-4075-243X

Olga Kozhar: https://orcid.org/0000-0002-0976-7334

Kristi Wharton: https://orcid.org/0009-0000-0847-1366

Jane E. Stewart: https://orcid.org/0000-0001-9496-6540

^1^To whom correspondence may be addressed: Carolyn Cornell, [carol.r.cornell@gmail.com](mailto:carol.r.cornell@gmail.com), Jane Stewart, [jane.stewart@colostate.edu](mailto:jane.stewart@colostate.edu), and Rabiu Olatinwo, [rabiu.o.olatinwo@usda.gov](mailto:rabiu.o.olatinwo@usda.gov)

**Keywords:** Brown spot needle blight, fungal pathogens, loblolly pine, climate variability, phyllosphere, endophytes

**SUPPLEMENTAL TABLES AND FIGURES**

| **Table S1** Commercial loblolly pine field study sites in central Louisiana | | | | | | | | |
| --- | --- | --- | --- | --- | --- | --- | --- | --- |
| **Site** | **Parish** | **Planted** | **Family** | **Soil type**^a^ | **Avg height (m; 2023)** | **Avg DBH (cm; 2023)** | **Avg height (m; 2024)** | **Avg DBH (cm; 2024)** |
| Site 1 (S1) | Vernon | 2014 | CMP1 | Malbis fine sandy loam | 6.7 | 11.2 | 8.5 | 11.9 |
| Site 2 (S2) | Vernon | 2018 | CMP2 | Eastwood silt loam | 3.8 | 4.1 | 4.6 | 5.8 |
| Site 3 (S3) | Rapides | 2018 | CMP3 | Ruston fine sandy loam | 3.4 | 3.6 | 3.7 | 4.5 |
| Site 4 (S4) | Rapides | 2017 | CMP4/OP1 | Ruston fine sandy loam/Malbis fine sandy loam | 7.2 | 12.2 | 9.1 | 13.7 |
| Site 5 (S5) | Rapides | 2018 | OP1 | Cahaba fine sandy silt loam | 5.8 | 9.7 | 7.8 | 11.5 |
| Site 6 (S6) | Rapides | 2017 | CMP4 | Caddo silt loam | 6.4 | 10.5 | 9.3 | 11.6 |

^a^Soil type based on NRCS soil reports (<https://websoilsurvey.nrcs.usda.gov/app/>)

**Table S2** Distribution of samples across sites, years, needle age classes, and symptom categories

|  | **October 2023** | | | | **October 2024** | | | |
| --- | --- | --- | --- | --- | --- | --- | --- | --- |
|  | **First-year** | | **Second-year** | | **First-year** | | **Second-year** | |
|  | **ASYM** | **SYM** | **ASYM** | **SYM** | **ASYM** | **SYM** | **ASYM** | **SYM** |
| **Site 1** | 10 | 10 | 10 | 10 | 10 | 10 | 10 | 10 |
| **Site 2** | 9 | 10 | 1 | 4 | 10 | 10 | 9 | 9 |
| **Site 3** | 10 | 10 | 0 | 0 | 10 | 10 | 8 | 10 |
| **Site 4** | 10 | 10 | 5 | 5 | 10 | 10 | 10 | 10 |
| **Site 5** | 10 | 10 | 4 | 4 | 10 | 10 | 4 | 9 |
| **Site 6** | 10 | 10 | 1 | 1 | **7** | 10 | 7 | 8 |

| **Table S3** Change in crown dieback between October 2023 to October 2024 | | | | |
| --- | --- | --- | --- | --- |
| **Site** | **Unchanged** | **Improved** | **Declined** |  |
| S1 | 8 | 2 | 0 |  |
| S2 | 7 | 0 | 3 |  |
| S3 | 6 | 4 | 0 |  |
| S4 | 7 | 1 | 2 |  |
| S5 | 4 | 0 | 6 |  |
| S6 | 7 | 1 | 2 |  |
| Trees were crown dieback level stayed the same from Oct 2023 to Oct 2024 are considered unchanged.  Ten trees were sampled at each site. | | | | |

**Fig. S1** Long-term drought severity in Central Louisiana. Standard precipitation index (SPI) at 1-year timescales between 1980 and 2025 in Rapides Parish, LA, and Vernon Parish, LA. SPI represents different levels of drought, including: extremely wet (> 2.00), very wet (1.50 to 1.99), moderately wet (1.00 to 1.49), normal (-0.99 to 0.99), moderate drought(-1.49 to -1.00), severe drought (-1.99 to -1.50), and extreme drought (< -2.00). Data collected from Climate Toolbox (<https://climatetoolbox.org/>).

**Fig. S2** Crown dieback differences across loblolly plantation sites. (a) Variation in diameter at breast height (DHB) with increasing levels of crown dieback. Crown dieback 1 represents low defoliation (< 30%), and crown dieback 3 represents high defoliation (< 90%). Box plot center lines represent the median values with whiskers extending to the minimum and maximum values. (b) Average crown dieback by loblolly plantation sites across sampling years. Sampling dates shown in gray (October 2023) and black (October 2024).

**Fig. S3** Fungal needle richness and diversity. Alpha diversity based on richness by (a) symptom status for first- (Year 1) and second-year (Year 2) needles. Richness of (b) first- and (c) second-year needles by crown dieback level. Crown dieback 4 (dead) samples were excluded from analysis due to the small sample size. Groups sharing the same letter were not significantly different. Significance expressed as *****P* < 0.0001. 3D principal coordinate analysis (PCoA) of Bray-Curtis dissimilarity of needles by year, broken up by (d) symptom status and (e) sampling year. Asymptomatic abbreviated as ASYM, and symptomatic abbreviated as SYM.

**Fig. S4** Difference in the relative abundance of abundant fungal genera in second-year needles. The relative abundance of dominant fungal genera by crown dieback level in second-year needles. Crown dieback 4 (dead) samples were excluded from analysis due to the small sample size. Asymptomatic abbreviated as ASYM.
